# Deoxynivalenol in food and feed: Recent advances in decontamination strategies

**DOI:** 10.3389/fmicb.2023.1141378

**Published:** 2023-03-14

**Authors:** Yingfeng Li, Huihui Gao, Ru Wang, Qing Xu

**Affiliations:** School of Food Science and Pharmaceutical Engineering, Nanjing Normal University, Nanjing, China

**Keywords:** deoxynivalenol, biodegradation, adsorption, enzymolysis, detoxification mechanism

## Abstract

Deoxynivalenol (DON) is a mycotoxin that contaminates animal feed and crops around the world. DON not only causes significant economic losses, but can also lead diarrhea, vomiting, and gastroenteritis in humans and farm animals. Thus, there is an urgent need to find efficient approaches for DON decontamination in feed and food. However, physical and chemical treatment of DON may affect the nutrients, safety, and palatability of food. By contrast, biological detoxification methods based on microbial strains or enzymes have the advantages of high specificity, efficiency, and no secondary pollution. In this review, we comprehensively summarize the recently developed strategies for DON detoxification and classify their mechanisms. In addition, we identify remaining challenges in DON biodegradation and suggest research directions to address them. In the future, an in-depth understanding of the specific mechanisms through which DON is detoxified will provide an efficient, safe, and economical means for the removal of toxins from food and feed.

## 1. Introduction

Over thousands of years, cereal grains emerged as an essential source of calories for humans and farm animals. In developed, developing, and poor countries, the proportion of energy provided by cereals to residents are ~30, >60, and >80%, respectively (Sidhu et al., 2007; Tsuge, 2014). Thus, the availability of safe dietary grains represents a cornerstone of human civilization (Awika, 2011). In 2016, the International Agency for Research on Cancer (IARC) and the World Health Organization (WHO) reported that mycotoxin pollution seriously affects the economic development and health of residents in developing countries (Weber et al., 2023), with approximately 500 million individuals in developing countries directly and indirectly exposed to mycotoxins every day (Fellone, 2016). A recent survey on mycotoxin contamination showed that in most of the tested food samples, the concentration of deoxynivalenol (DON) exceeded the recommended limits (Mishra et al., 2019).

**Figure 1:**
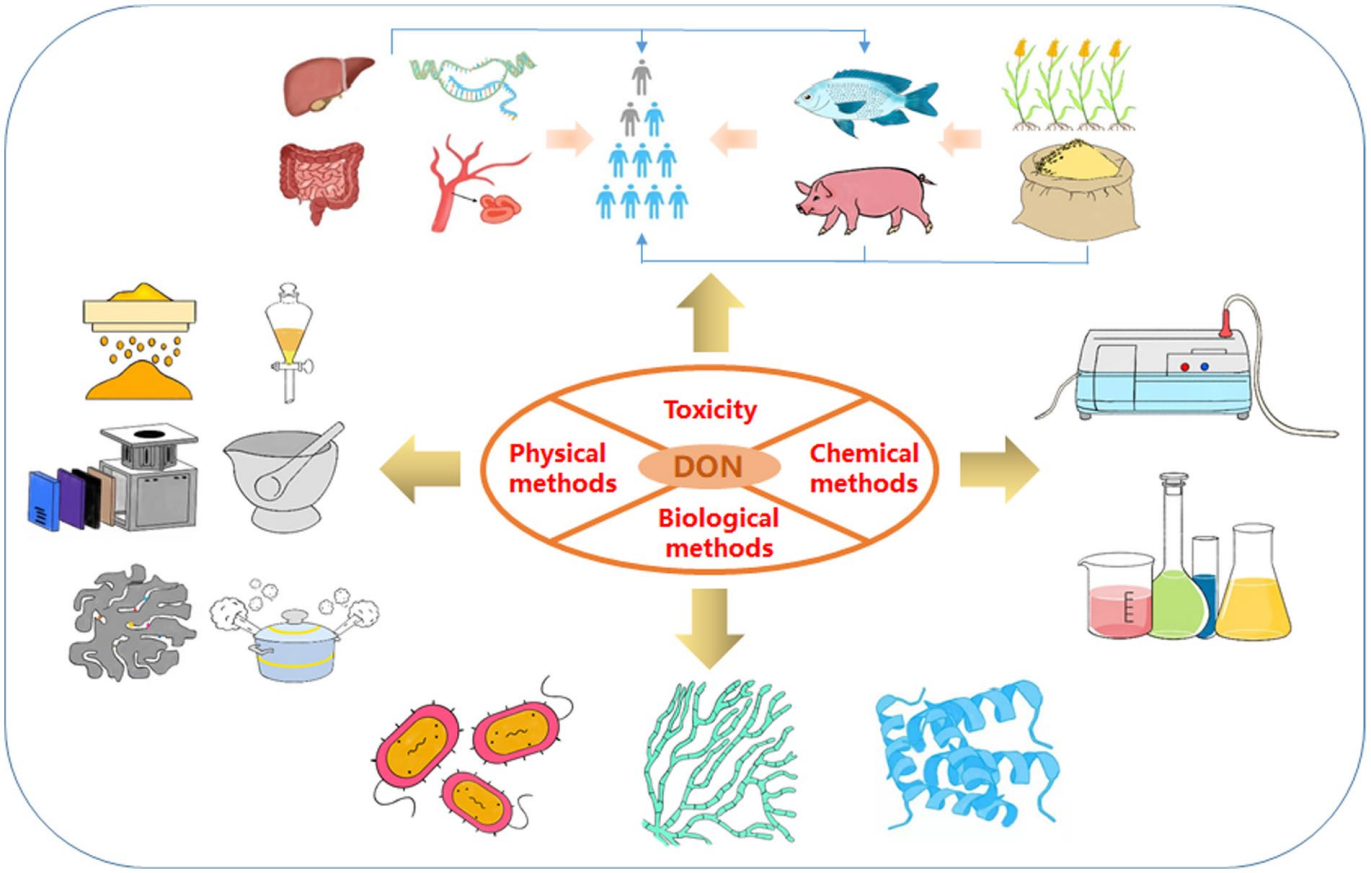
Framework of the review. These include the toxicity of DON and three detoxification methods (physical methods, chemical methods and biological methods).

DON, also known as vomitoxin, is a type B trichothecene and epoxy-sesquiterpenoid produced by *Fusarium graminearum*. It was first isolated and purified from moldy barley grains by Yoshizawa et al., and chemically characterized in 1970 (Han et al., 2020). DON is soluble in water, ethanol, acetonitrile, and other polar solvents. It is also stable at high temperatures and low pH. Previous studies have shown that wheat contaminated with DON is still toxic after 4 years of storage (Cao et al., 2013). DON can contaminate food at any step of food production, including processing, transportation, storage, and consumption, generally by contaminating plant products, directly or indirectly contaminating food and animal feed, and finally stably accumulating in the human food supply (Figure 1; Zhai et al., 2019a).

**Figure 2:**
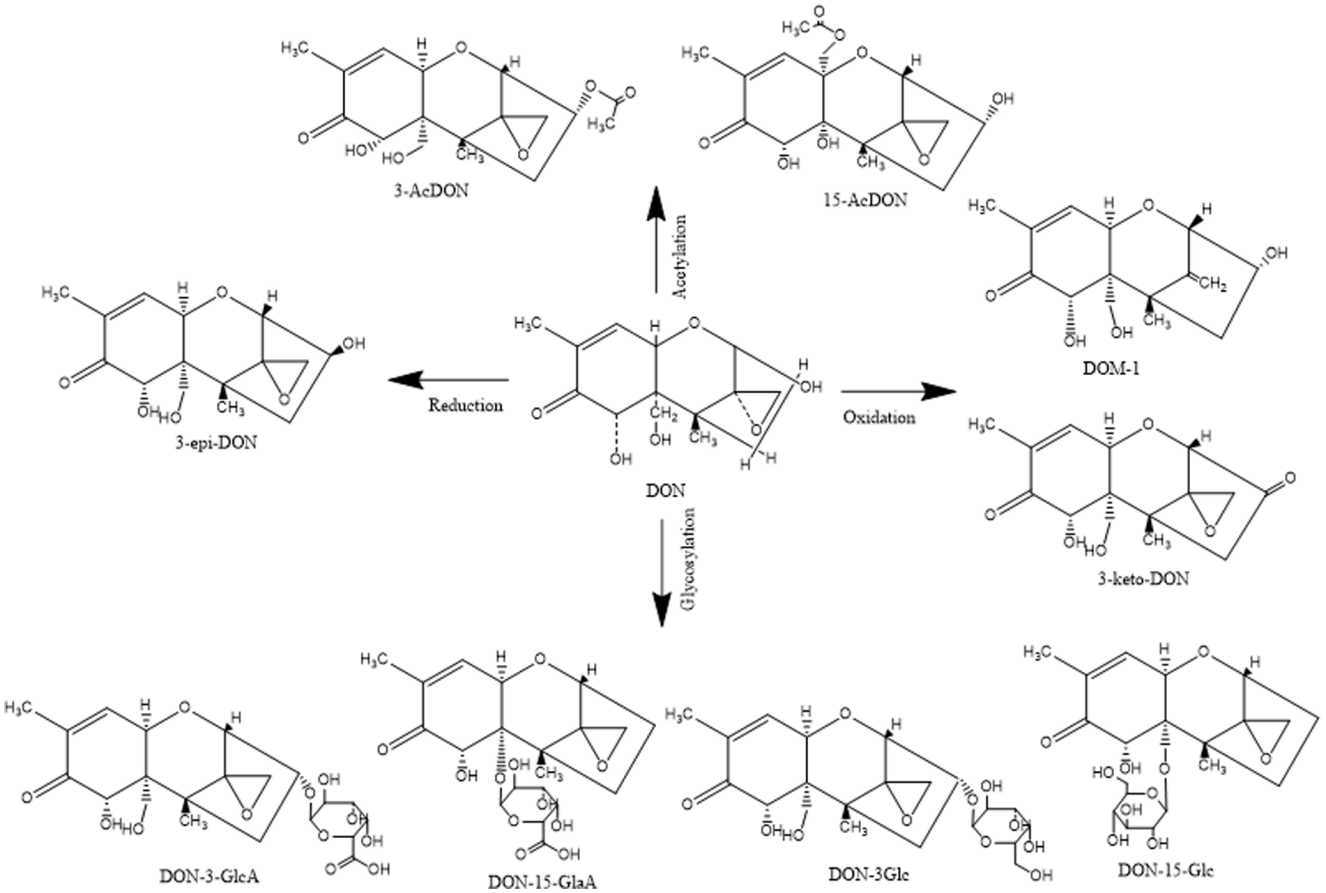
Structural formula of DON and its derivatives.

In addition, DON derivatives may be present in food and animal tissues such as blood, muscles, and liver, including acetylated (3-ADON and 15-ADON), redox modified (3-keto-DON, DOM-1, and 3-epi-DON), and glycosylated (D3G and D15G) forms of DON, as well as glucuronic acid derivatives (D3GA and D15GA; Figure 2). DON and its derivatives share a common epoxide group at C12–13, which is also a key group in determining their toxicity (Pestka et al., 2004). Epoxy compounds can damage cells by inhibiting the activation of various protein kinases, dysregulating gene expression, and inhibiting protein synthesis (Pestka, 2010).

DON mainly contaminates starchy staple foods such as wheat, barley, oats, rye, corn, and potatoes, among which wheat, corn, and oats are the most polluted (Golge and Kabak, 2020; Topi et al., 2021). Correspondingly, many countries have formulated highest acceptable limits for DON in grains, according to their national circumstances. For instance, the national standard of China, GB2761-2017, stipulates that the standard limit for DON in grain and grain products is 1 mg/kg (China, 2017). Similarly, the Food and Drug Administration of the United States stipulates that the safety standard for DON in food is 1 mg/kg, while the allowable limit in wheat and wheat products in the United States must not exceed 4 mg/kg (Feizollahi and Roopesh, 2022). By contrast, the content of DON in grain flour and corn in the European Union must be <0.75 mg/kg, and this limit is relatively strict. Therefore, there is an urgent need to remove DON from food products and animal feed.

To date, most reviews only focused on discussing decontamination methods for several kinds of mycotoxins, while there is still no in-depth and comprehensive summary and discussion of DON detoxification. Nevertheless, DON contamination has had a great impact on food safety in recent years, and many new decontamination approaches have been investigated to solve the challenge of DON detoxification. In this review, we discuss three recent approaches to DON detoxification, which include physical, chemical, and biological methods (Figure 1). Through a comprehensive literature search and analysis, it was found that traditional DON detoxification strategies, including physical and chemical approaches, often have disadvantages, while biological detoxification approaches have the advantages of no secondary pollution, high efficiency, and specificity. Chemical detoxification methods include the structural degradation of aldehydes, oxidants, acids, bases and several gases. However, chemical treatment usually affects the palatability and nutrition of feed and food, and is not suitable for large-scale detoxification. In addition, it is also easy to cause secondary pollution. Biological methods include the use of bacteria, yeast or their respective enzymes or metabolic pathways to degrade DON. Especially, enzymatic detoxification technology has good repeatability, uniformity, simple operation and high safety, as well as some other interesting properties. Therefore, enzymatic degradation is considered to be the most commercially valuable and useful technology in future applications. This review provides a theoretical basis for the development and practical application of DON decontamination technologies.

## 2. Strategies for the nonbiological detoxification of DON

### 2.1. Physical methods

Physical detoxification includes screening and stripping, washing and grinding, radiation treatment, heat treatment, and adsorption. According to the different mechanisms of action, physical detoxification methods can be divided into approaches based on either the intact removal or the degradation of DON.

#### 2.1.1. Removal of DON

The physical removal of intact DON consists of separating the contaminated outer coat and pericarp of the kernels *via* procedures usually used in cereal processing, such as separation, solvent extraction, and adsorption.

##### 2.1.1.1. Peeling and grinding

Mycotoxins tend to be concentrated in the germ and bran of grains (Brera et al., 2004). In the early stages, DON contamination in grains can be removed by mechanical peeling. Trenholm et al. removed the fungal chitin from barley by coarse grinding and sieving, reducing the DON content by 16%. However, the mass loss of barley, wheat, and corn reached 34%, 55%, and 69% of the total matter, respectively, while nearly 40% of the ground grain was lost. Moreover, 22%–32% of the protein was also lost (Trenholm et al., 1991). This method can only remove cereal grains, broken grains, and fine substances with high mold contamination, and does not completely eliminate toxin contamination. Furthermore, cereal grains are more likely to be invaded by mycotoxigenic fungi after mechanical damage (Magan et al., 2003).

##### 2.1.1.2. Solvent extraction

DON is soluble in water and polar solvents, so it can be extracted, isolated, and detoxified by a mixture of water and organic solvents. The common extraction solvents reported in the literature include distilled water, saturated sodium chloride solution, 30% sucrose solution, sodium carbonate, and sodium hydroxide (Yener and Köksel, 2013). These extractants can eluate the DON present in food to a certain extent, but these methods also have some disadvantages. For example, rinsing will leave DON and its by-products in the solution, resulting in secondary contamination. Moreover, there are limitations such as high cost and the need for large-scale processing equipment. In addition, the nutritional value of the grains is reduced during the extraction process.

##### 2.1.1.3. Material adsorption

As shown in Table 1, this process functions by adsorbing the toxin to a foreign body composed of various adsorbent materials, such as layered silicate minerals, activated carbon, nano zeolite, or synthetic resin (Huwig et al., 2001). Montmorillonite (MT) is a complex clay mineral that has been proven to be an efficient adsorbent. Among the different types, pillared montmorillonite has aroused great interest because of its large surface area and catalytic properties. In 2021, Zhang et al. synthesized three types of pillared montmorillonite containing Al, Fe, and Ti. In their DON adsorption experiments, it was found that the adsorption capacity of pillared montmorillonite was 3–4 times higher than that of the original montmorillonite. However, this material works in a low acidic environment, which is not conducive to maintaining the original nutritional properties of food (Zhang et al., 2021). In recent years, new functional microspheres have been synthesized for the adsorption of mycotoxins. For example, Shalapy et al. first used alginate/sodium hydroxylmethyl cellulose composite calcium (SA/CMC-Ca) to form functional microspheres for the adsorption of 3.6 mg/L DON from contaminated corn leachate (Shalapy et al., 2020).

**Table 1:** Recent studies on material adsorption for DON removal.

Treatment	DON concentration	Main results	Mechanism	References
Activated carbon	5 mg/kg	AC minimizes adsorption of toxins from the gastrointestinal tract	Adsorption	Abdel-Wahhab et al. (2015)
Cross-linked chitosan polymers	1 μg/mL	≤30%	Adsorption	Zhao et al. (2015)
Pillared montmorillonite	500 ng/mL	35%	Adsorption	Zhang et al. (2021)
Microsphere Adsorbent SA/CMC Loaded with Calcium	3.6 μg/mL	35%	Adsorption	Shalapy et al. (2020)
Ion-exchanged zeolites	2 mg/mL	37%	Adsorption	Savi et al. (2018)
Modified nano-montmorillonite (NMMT-STAB)		Equilibrium adsorption capacity of 2.13 mg/g	Adsorption	Zhang W. et al. (2020)

As countries roll out sustainable development strategies, some researchers are turning toward the use of renewable materials for DON detoxification. For example, humic acid (HA), durian peel (DP), sodium alginate (SA), and other substances can adsorb DON (Adunphatcharaphon et al., 2020; Haus et al., 2020; Zhang L. et al., 2020). However, these adsorbents not only have low adsorption efficiency but are also expensive (Shehata et al., 2000).

#### 2.1.2. Degradation of DON

The physical degradation of DON mainly consists in destroying its chemical bond to form new non-toxic or less toxic compounds. The methods are mainly divided into heat and non-heat treatments.

##### 2.1.2.1. Heat treatment

This process can detoxify mycotoxins produced by fungi growing on grains (Bullerman and Bianchini, 2007). Heat treatment breaks the chemical bonds of DON at high temperatures to achieve detoxification. In the 1980s, methods were developed to reduce the toxicity of DON by high-temperature treatments, such as steaming, boiling, and frying (El-Banna et al., 1983; Scott et al., 1984). Bretz et al. heated DON under alkaline conditions and analyzed the decomposed products by nuclear magnetic resonance (NMR) and mass spectrometry (MS), revealing that DON was transformed into several new compounds with less toxicity (Bretz et al., 2006). However, the thermal stability of DON makes it resistant to the high temperatures encountered during food processing. Some studies have shown that DON detoxification in food is non-reversible only when the treatment temperature is above 150°C. However, high temperature treatment leads to the loss of some nutrients in food, and may produce toxic or unpalatable by-products that affect the taste (Humpf et al., 2004; Bullerman and Bianchini, 2007).

##### 2.1.2.2. Non-heat treatment

In addition to heat, physical treatments such as ultraviolet light, pulsed light technology, and atmospheric cold plasma (ACP) can also destroy mycotoxins on the surface of food and materials (Elmnasser et al., 2007; Karaca et al., 2010; Murata et al., 2011; Hojnik et al., 2019). For example, Stepanik et al. reported that wheat contaminated with DON was irradiated to doses ranging from 2.0 to 55.8 kGy by an electron accelerator, and found that electron beam treatment could reduce 17.6% of DON level in wheat at the highest dose used (55.8 kGy; Stepanik et al., 2007). Li et al. used ^60^Co γ-ray irradiation to degrade DON and found that 20 kGy γ-ray irradiation could degrade 83% of DON in acetonitrile–water, while 2 μg/mL DON in ultra-pure water was completely degraded after 5 kGy γ-ray irradiation (Li et al., 2019a). Murata et al. used ultraviolet (UV) light (1.5 mW/cm^2^ at 254 nm UV-C wavelength), which removed 22% of the initial concentration of 60 mg/kg DON within 30 min without affecting the total content of vitamins (Murata et al., 2011). However, this method is not only inefficient, but also greatly affects the taste and nutrient composition of flour. In 2013, Moreau et al. found that pulsed light treatment could remove 72.5% of DON, but the residual concentration was still toxic in *Caenorhabditis elegans* survival tests (Moreau et al., 2013). Moreover, light generally does not penetrate solid foods such as grains, and these methods are often only suitable for liquid or transparent objects. ACP has been used to chemically decompose mycotoxins through various pathways, resulting in degradation products of low toxicity (Misra et al., 2019). In 2017, Ten Bosch et al. evaluated the effect of ACP technology on the degradation of several mycotoxins. The experimental data showed that DON was almost completely degraded after 60 s and nontoxic residue (Ten Bosch et al., 2017). ACP technology is expected to be applied in the degradation of the DON present in food, but the underlying mechanism remains unknown.

## 3. Chemical methods

Chemical detoxification uses strong oxidizing agents, strong acids and bases, or ozone to detoxify grains contaminated with DON. This process converts DON into degradation products with low or no residual toxicity.

### 3.1. Ozonization

This new process, which was only recently approved for application in the food industry, has achieved good results in the detoxification of contaminated products, offering an effective and safe chemical method for the reduction or elimination of mycotoxins in grains (Karaca et al., 2010; Tiwari et al., 2010). The DON degradation percentages in ultrapure water and acetonitrile solution after treatment with gaseous ozone reached 95.68% and 98.3%, respectively, and there were no significant degradation products (Li et al., 2019b; Ren et al., 2020). However, the direct effect of ozone on the removal of DON from grains is not obvious. Wang et al. used gaseous ozone to treat a DON-containing solution and *F. graminearum*-infected wheat. The results showed that 10 mg/L ozone could degrade 93.6% of the DON present in the solution within 30 s, but treating the infected wheat with 80 mg/L ozone for 2 h could only remove 26.11% of the DON found in the grains. The double bond of DON structure was destroyed by ozone, and the toxicity of the product should be weaker than that of DON. Furthermore, high-moisture wheat was more sensitive to ozone than low-moisture wheat under the same conditions. The effect of ozone on DON was higher in the solution than in the cereal grains, possibly because the specific grain morphology educed the contact of ozone with DON. Therefore, Wang et al. explored the effect of ozone on the degradation of DON and its derivatives in wheat with different water contents and raw material morphologies, using different ozone concentrations, exposure times. It was found that the DON degradation percentage increased significantly with an increase of ozone concentration and exposure time. In addition, DON was degraded more easily in whole wheat flour than in wheat grains, and the decrease of DON content became more significant as the humidity increased (Wang et al., 2016). These studies offer a strong theoretical basis for the future ozone-based removal of DON contamination from grains. The application of ozone for grain storage and preservation is presented in Table 2. In addition to ozone, ammonia (NH3), chlorine (Cl2), and other gases can also detoxify the DON present in grains (Young, 1986; Borràs-Vallverdú et al., 2020).

**Table 2:** Application of ozone in grain storage and preservation.

Treatment	Type of products	DON concentration	Assay conditions	Detoxification (%)	Mechanism	References
Ozone	Wheat	1,000 μg/g	Moist ozone (1.1 mol %) for 1 h	90%	Degradation	Young (1986)
Ozone	maize	-	50 ppm for 3d	63%	Degradation	Kells et al. (2001)
Ozone	DON in acetonitrile solution	50 mg/L	80 mL/min for 9 min	98.3%	Degradation	Ren et al. (2020)
Ozone	DON solution	1 μg/mL	10 mg/L for 30s	93.6%	Degradation	Li M. M. et al. (2015)
Ozone	DON solution	2 μg/mL	8 mg/mL for 15 s	95.68%	Degradation	Li et al. (2019a,b)
Ozone	Wheat	100 mg/L	3.89 mg/kg	78.7%	Degradation	Wang et al. (2016)

### 3.2. Sodium treatment

Although ozonization is effective, the process requires specialized equipment and is costly, which makes it currently unsuitable for large-scale use (Li M. M. et al., 2015). Chemical detoxification involves the use of specific chemicals to destroy the chemical structure of mycotoxins or to neutralize their active groups (Wu et al., 2020). For example, sodium metabisulfite (SMBS) can destroy DON in processed grains or feed. Danicke et al. conducted a series of experiments using SMBS to determine whether the content of DON in cereals can be effectively reduced by controlling the concentration of SMBS under hot water treatment (Danicke et al., 2005, 2009, 2010). SMBS can rapidly degrade DON under acidic aqueous conditions (such as in the pig stomach; Lu et al., 2020).

Although physical and chemical detoxification methods are relatively simple, they also have many drawbacks. For example, the toxin removal efficiency is low, the treatment cost is high, it often destroys various nutrients in grains, and the degradation products can also be toxic.

## 4. Biological methods

These are considered promising detoxification methods because they are environmentally friendly, require mild reaction conditions, show strong specificity and have high efficiency (McCormick and Susan, 2013). These methods mainly rely on the adsorption of DON onto microbial cell walls or the degradation of DON by microbial enzymes.

### 4.1. Biosorption of DON

The cell walls of certain bacteria can intrinsically adsorb mycotoxins due to their specific composition. For example, the cell wall contains carbohydrates (peptidoglycan, mannose, glucose proteins, and lipids), whose adsorption mechanisms may include hydrogen bonds, ion interactions, and/or hydrophobic interactions (Chen et al., 2018; Wang N. et al., 2019; Chlebicz and Slizewska, 2020).

In recent years, yeast has been used as a nutritional additive and added to feed as an antidote in the actual production process. The addition of *Saccharomyces cerevisiae* to a PBS buffer containing DON was shown to decrease the soluble DON content by 39% (Chlebicz and Slizewska, 2020). Lactic acid bacteria (LAB) can inactivate mycotoxins or convert them into less toxic products by adsorbing them and forming mycotoxin-organic adsorption complexes. Experiments have shown that the cell wall of *Lactobacillus paracasei* LHZ-1 can form a complex with DON, reducing its content in solution by 40.7% within 24 h (Zhai et al., 2019b). Table 3 lists studies on the adsorption of DON by microorganisms.

**Table 3:** Study on the adsorption of DON by microorganisms.

Strain	Removal rate(%)	Source
*Rhodotorula rubra*	47.7%	Bakutis et al. (2005)
*Rhodotorula glutinis*	84.6%	
*Metschnikowia pulcherima*	55%	
*Geotrichum fermentans*	62.7%	
*Kluyveromyces marxianus*	44%	
*Lactobacillus rhamnosus* GG	18%–93%	El-Nezami et al. (2002)
*Lactobacillus plantarum*	55%	Niderkorn et al. (2006) and Zou et al. (2012)
*Aspergillus oryzae*	74%	Garda-Buffon and Badiale-Furlong (2010)
*Rhizopus oryzae*	90%	
*Streptococcus thermophilus*	33%	Niderkorn et al. (2007)
*Lactobacillus rhamnosus* GG	54%	Niderkorn et al. (2006)
R0011	30%	
*Saccharomyces cerevisiae strains*	19%–39%	Chlebicz and Slizewska (2020)
*Lactobacillus paracasei* LHZ-1	40.7%	Zhai et al. (2019b)

Furthermore, the mycelia of some filamentous fungi can absorb large amounts of DON. For example, in a study on submerged fermentation, Garda-Buffon et al. found that the hyphae of *Rhizopus oryzae* and *Aspergillus oryzae* can adsorb DON, whereby the highest removal efficiency reached 74 and 90% after agitation for 96 and 240 h, respectively (Pierron et al., 2016).

### 4.2. Biodegradation

Biodegradation and detoxification occur mainly through the destruction of toxic groups and the conversion of DON into less toxic or non-toxic compounds by microorganisms or enzymes (Guo et al., 2020). Some examples of the resulting substances are 3-keto-DON, 3-epi-DON, and 3-oxo-DON, whereby the toxicity of 3-keto-DON is 1/10 than that of DON, and that of 3-epi-DON is 1/1181 (Shima et al., 1997; Karlovsky, 2011; He et al., 2015; Pierron et al., 2016).

#### 4.2.1. Degradation of DON by microorganism

Many microorganisms, including bacteria, fungi, actinomycetes, and algae, can remove and degrade mycotoxins from food and feed (Hathout and Aly, 2014).

##### 4.2.1.1. Detoxification using bacteria

As shown in Table 4, a number of bacteria degrade DON, including several species of the genus *Bacillus*. The DON degradation percentages after treatment with *Bacillus subtilis* ZZ and *B. licheniformis* reached >98 and 72.2%, respectively (Cheng et al., 2010). *Bacillus cereus* isolated from moldy straw, soil, and feces can also degrade DON (Ergang, 2016). Moreover, Tan et al. screened the *B. subtilis* strain NHIBC006D from contaminated corn and found that it could degrade 73.5% of DON (Tan et al., 2018).

**Table 4:** Degradation of DON by different microorganisms.

Strain	Degradation products	Degradation rate	Source
*Devosia muans* 17-2-E-8	3-epi-DON		He et al. (2016)
*Bacillus cereus* B.JG05	-	>80.61%	Ergang (2016)
*Bacillus subtilis*	-	73.5%	Tan et al. (2018)
*B. subtilis* ZZ	-	>98%	Cheng et al. (2010)
*B. licheniformis* DY		71.4%	
*Eggerthella sp.* DII-9	-	-	Gao et al. (2018)
*Slackia* sp. D-G6(D-G6)	DOM-1	100%	Gao et al. (2020)
*Agrobacterium–Rhizobium* strain E3-39	3-keto DON	-	Jun Shima et al. (1997)
*Nocardioides sp.* strain WSN05-2	3-epi-DON	90%	Ikunaga et al. (2011)
*Mixed flora*	-	70%	Junmei (2013)
ODS-1		96.2%	
*Devosia sulae* A16	3-keto-DON	88%	Wang G. et al. (2019)
*Lactobacillus rhamnosus* SHA113	3-epi-DON	60%	Qu et al. (2019)
*Lactobacillus rhamnosus* RC007	-	-	Romina Garcia et al. (2018)

Ikunaga et al. screened the *Nocardia* sp. strain WSN05-2 from the soil of a wheat field based on its ability to grow in a medium with DON as the sole carbon source. After 7 days of cultivation, the DON degradation ratio reached approximately 90%. The resulting metabolite was identified as 3-epi-DON by MS and ^1^H and ^13^C NMR analyses (Ikunaga et al., 2011). Another strain was also screened from the soil of a wheat field and identified as *Devosia insulae* by morphological, physiological, and 16S rRNA phylogenetic analyses. This strain was able to degrade 88% of 20 mg/L DON within 48 h under aerobic conditions and neutral pH at 35°C. Moreover, it was found that the main degradation product of DON and its derivatives was 3-keto-DON (Wang G. et al., 2019). Furthermore, they identified oxidation of the C-3 hydroxyl as the degradation mechanism. In addition, researchers tested the oxidative degradation of DON in animal intestines. For example, Yu et al. screened strains capable of transforming the DON present in chicken intestines using denaturing gradient gel electrophoresis (PCR-DGGE). Ten strains could convert DON into the less toxic deepoxy-4-deoxynivalenol derivative (DOM-1; Yu et al., 2010). Gao et al. the novel *Eggerthella* sp. strain DII-9 in chicken intestines. It was able to convert DON into another substance over a wide range of temperatures (20–45°C) and pH values (5–10; Gao et al., 2018). Later, the team also found a new DON detoxifying strain, *Slackia* sp. D-G6 in chicken intestines, which is a gram-positive non-sporulating bacterium that can de-epoxidize DON into DOM-1. Using this bacterium and a specific chicken intestinal extract medium, 25 mg/L DON was completely transformed into DOM-1 within 48 h (Gao et al., 2020).

##### 4.2.1.2. Detoxification using fungi

In addition to adsorbing mycotoxins, some fungi metabolize and degrade mycotoxins during fermentation, converting them into compounds with low or no toxicity. For example, yeast can convert DON into D3G, 15-A-DON, and 3-ADON (Freire and Sant’Ana, 2018). Nathanail et al. reported that *Saccharomyces pastorianus* converts DON into D3G by binding to DON glycosides during fermentation, whereby DON and D3G can also be adsorbed by the yeast cell wall (Nathanail et al., 2016). He et al. used DON as the sole carbon source and isolated a soul fungus that could transform DON, which was named NJA-1. The strain was identified as *Aspergillus tubingensis* by gene series analysis and morphological observations. The conversion rate of DON reached 94.4% within 14 days. The results of HPLC-MS analysis showed that the fluorescence intensity of the degradation products was 18.1 higher than that of DON. The highest tolerance concentration of NJA-1 to DON in an inorganic salt medium was 40 mg/l (He et al., 2008). Furthermore, strains of *Aspergillus oryzae* and *Rhizopus oryzae* were also found to adsorb and degrade DON. *Aspergillus oryzae* KKB4 and *Rhizopus oryzae* KP1R1 were inoculated into DON-contaminated corn and fermented for 10 days, which resulted in DON reduction by 65.91% and 56.82%, respectively. Toxicity analysis showed that the degradation products did not affect the growth of *Saccharomyces cerevisiae* (Arifin et al., 2019). Moreover, the DON degradation percentage in corn medium was as high as 92% after culture of *Aspergillus oryzae* (5509NRRL) for 21 days (Tran and Smith, 2014).

##### 4.2.1.3. Detoxification using microbial consortia

Microbial consortia are considered to offer special advantages for the degradation of complex compounds (Yuan et al., 2016). In the microbial consortium, different microorganisms cooperate with each other to achieve the best degradation effect. Researchers began to screen out bacterial and microbial complexes through selective culture and domestication for toxicity reduction of different compounds, including aflatoxin B1 (AFB1), ZEN, DON, and their derivatives (Wang et al., 2017; Zhai et al., 2019a; Wang et al., 2020a). For example, 10 bacterial cultures were screened from pig manure by Junmei et al., and the DON degradation percentage in the simultaneous degradation of ZEN, AFB1, and DON by multiple bacteria reached up to 70% (Junmei, 2013).

Wang et al. screened the bacterial consortium C20, which could degrade nearly 70 mg/l DON within 5 days, and confirmed the conversion of DON into 3-keto-DON by liquid chromatography/time-of-flight MS (LC-TOF-MS) and NMR analyses (Wang et al., 2020b). In the same year, Wang et al. screened a DON-degrading bacterial consortium from wheat leaves, which was transformed into 3-keto-DON through a two-step differential isomerization, as determined by metabolomics and NMR analyses. During the degradation of DON, it was found that the abundance of achromatic bacilli and *Sphingomonas* sp. in the flora increased greatly, and it was speculated that these two types of bacteria played a significant role in the degradation of DON (Wang G. et al., 2020). Zhai et al. reported for the first time the two-step enzymatic isomerization of DON in a mixed culture. They screened the bacterial consortium LZ-N1, which showed high efficiency and stable DON transformation activity in medium with DON as the sole carbon source. High-throughput analysis showed that LZ-N1 was composed of at least 11 bacterial species, among which *Pseudomonas* sp. accounted for nearly half of the relative abundance. When two of the newly identified strains, *Pseudomonas* sp. Y1 and *Lysobacter* sp., were co-cultured, it was found that cell-free supernatant, intracellular lysate, and cell fragments had the ability to degrade DON. When the concentration of DON was 50 mg/L, the degradation in the supernatant could reach 100% after 48 h (Zhai et al., 2019b). Although microbial consortia have good degradation effects, the community often becomes unstable during degradation. Furthermore, there is still no in-depth investigation of the microbial dynamics of such consortia.

#### 4.2.2. Degradation of DON by enzymes

**Figure 3:**
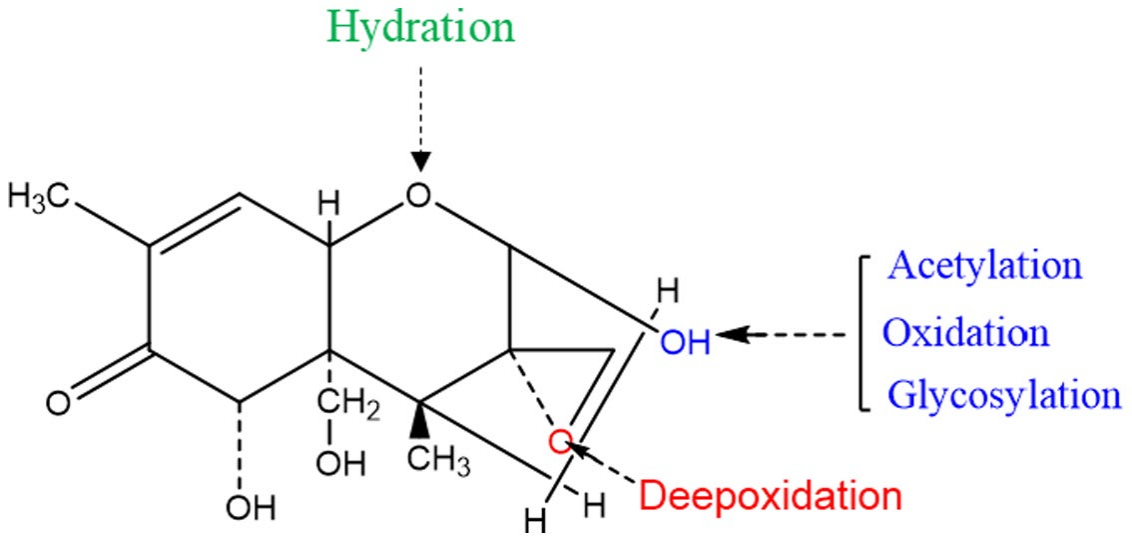
Detoxification of the target toxic group in the DON structure.

Certain enzymes or proteins produced by microorganisms can transform or bind mycotoxins. As shown above, many microorganisms can convert DON by metabolizing it with specific enzymes. Because enzymatic degradation has the advantages of high specificity, efficiency, and environmental friendliness, studies are increasingly focusing on the identification, purification, and characterization of detoxifying enzymes, as well as the underlying catalytic mechanisms. According to the literature, the mechanisms of enzymatic DON detoxification can be divided into acetylation, oxidoreductase and glycosylation (Table 5). The sites of action of these enzymes are shown in Figure 3.

**Table 5:** DON degradation enzymes and their products.

Mechanism	Degradation product	Degrading enzyme/gene	Reaction type	References
Destroys the epoxy structure at C12–C13	DOM-1	/	Oxidordeuctase	Yu et al. (2010)
Oxidizes the hydroxyl group on C3 to a ketone group	3-keto-DON	3-AcetylDON Oxidase		Zhang L. et al. (2020)
	3-oxo-DON	DepA enzyme/AKR18A1		He et al. (2017)
Isomerizes C3	3-epi-DON	DepB enzyme		He et al. (2016)
Hydrogenates DON	16-HDON	DdnA (cytochrome P450 system)		Ito et al. (2013)
C3–OH acetylated form of DON	3-ADON/15-ADON	3-O-acetyltransferase	Acetylation	Hao et al. (2021)
UDP-glycosyltransferase	3-β-D-glucopyranosyl4-DON	Glycosylation, enhancing the resistance of Arabidopsis thaliana to DON	Glycosylation	Poppenberger et al. (2003)
HvUGT13248	D3G	Glycosylation, enhancing the resistance of wheat to DON		Li X. et al. (2015)

##### 4.2.2.1. Acetylation

DON, nivalenol (NIV), and 3,7,15-trihydroxy-12,13-epoxytrichothec-9-ene (NX-3) are produced by *Fusarium graminearum*. Hao et al. explored the use of fungal self-protection agents in plants and found that *Fusarium graminearum* trichothecene 3-O-acetyltransferase (FgTri101) can degrade DON, 15-ADON, NIV, and NX-3. They cloned FgTri101 from *Fusarium graminearum* and expressed it in *Arabidopsis* plants. The experimental results confirmed that FgTri101 transgenic plants can acetylate DON to 3-ADON, as well as 15-ADON to 3,15-diDON (Hao et al., 2021). Furthermore, Wang et al. found that 3-ADON and 15-ADON can be deacetylated to produce DON, which is then degraded into 3-keto-DON (Wang N. et al., 2019).

##### 4.2.2.2. Oxidoreductases

The C12-C13 epoxy structure in DON is the key determinant of its toxicity, and its conversion is accordingly crucial for degradation. Studies have found that resistance genes or secreted proteins in some microorganisms destroy the epoxy structure of DON. For example, *Bacillus* sp. LS100 can destroy the epoxy structure, thereby degrading DON into DOM-1 (Li et al., 2011). In addition, the C3 hydroxyl group of DON can be oxidized to generate the corresponding ketone group. Zhang et al. screened a new strain, *Pelagibacterium halotolerans* ANSP101, which contains a protein that can oxidize the C3 hydroxyl group and convert DON into the less toxic, 3-keto-DON (Zhang J. et al., 2020). The C3 hydroxyl group of DON was isomerized to form 3-epi-DON. This isomerization requires two enzymatic actions: the conversion of DON into 3-oxo-DON, and that of 3-oxo-DON to 3-epi-DON (Hassan et al., 2017). In addition, resistance genes have also attracted much attention in studies on the degradation of DON. Ito et al. used the KSM1 gene library to screen out that the P450 gene (*ddnA*), is part of an electron transport chain *in vitro* that decomposes DON into the less toxic, 16-hydroxy-deoxynivalenol (16-HDON), thereby reducing the harm to wheat and other grains (Ito et al., 2013).

##### 4.2.2.3. Glycosylation

Poppenberger et al. discovered that the UDP-glycosyltransferase from *Arabidopsis thaliana* can catalyze the transfer of glucose from UDP-glucose to the C3 hydroxyl group of DON to form 3-β-D-glucopyranosyl-4-DON. The overexpression of this enzyme increases the resistance of *Arabidopsis thaliana* to DON (Poppenberger et al., 2003). Later, it was found that the overexpression of HvUGT13248 (UDP glycosyltransferase) could improve the resistance of wheat and *Brachypodium distachyon* to DON (Li X. et al., 2015; Pasquet et al., 2016).

##### 4.2.2.4. Other enzymes

In addition to the DON-converting enzymes mentioned above, there are also some DON degradation processes and degradation products that are still not fully understood. For example, Gardiner et al. analyzed the RNA profile of barley ears treated with DON and found that glutathione-S-transferase gene transcription was significantly upregulated (Gardiner et al., 2015). JuoDeIkiene et al. found that the combination of β-xylanase and traditional amylolytic enzymes can degrade 41% of DON at high concentrations (3.95 mg/kg), and can improve distillers dried grains with solubles without affecting the quality of bioethanol (JuoDeIkiene et al., 2011).

Enzymatic detoxification technology has the characteristics of good repeatability, uniformity, simple operation, and high safety. Therefore, enzymatic degradation is considered the most commercially viable and promising technology. However, the enzymatic hydrolysis of DON is still in its infancy, and there is still a long way to the discovery and optimization of new degradation enzymes. Adding to the challenge, the purification of detoxification enzymes is a time- and labor-consuming process, while the large-scale application of natural enzymes is limited because of the easy deactivation of natural enzymes in industrial environments such as strong acids, strong alkalis, and high temperatures. Therefore, cloning new DON-degrading enzyme genes is a promising approach. In addition, immobilizing enzymes and improving their physicochemical properties using biomaterials synthesized by biomimetic mineralization may also prove to be a worthwhile research direction.

## 5. Conclusion

DON is a widely distributed mycotoxin that frequently contaminates crops and animal feed. In contrast to traditional physical and chemical methods, biological methods can effectively convert DON into less toxic or even completely harmless products. However, there are still some problems that need to be solved: (1) the degradation mechanisms of many microorganisms and the toxicity of the degradation products remain unclear, which limits their application potential; (2) the biosafety and DON degradation pathways of most detoxifying bacteria have not been clarified; (3) there are still few studies on the cloning and functional expression of genes responsible for the DON detoxification phenotype. Therefore, an in-depth study on detoxification mechanisms is needed to provide reliable theoretical support for the application of detoxifying bacteria and enzymes. Increasing numbers of studies are focusing on genomic, transcriptomic, and proteomic analyses, which offer fast and efficient tools for the study of DON detoxification pathways as well as the cloning and expression of related genes. In the future, an in-depth understanding of the specific mechanisms through which DON is detoxified will provide efficient, safe, and economical means for the removal of this problematic mycotoxin from grains and feed.

## Author contributions

YL and RW: research literature and writing-original draft. HG: manuscript revision and literature review. QX: resources, investigation, supervision, conceptualization, and project administration. All authors contributed to the article and approved the submitted version.

## Funding

This work was financially supported by National Key Research and Development Program of China (No. 2020YFC1606800) and the China Postdoctoral Science Foundation (2021M691624).

## Conflict of interest

The authors declare that the research was conducted in the absence of any commercial or financial relationships that could be construed as a potential conflict of interest.

## Publisher’s note

All claims expressed in this article are solely those of the authors and do not necessarily represent those of their affiliated organizations, or those of the publisher, the editors and the reviewers. Any product that may be evaluated in this article, or claim that may be made by its manufacturer, is not guaranteed or endorsed by the publisher.
